# Application of laparoscopy, choledochoscopy, and duodenoscopy in the treatment of gallstones with choledocholithiasis

**DOI:** 10.3389/fsurg.2026.1752253

**Published:** 2026-06-22

**Authors:** Wei Wu, Xinhua Wu, Rixin Zhang, Xiaowan Li, Ting Li, Ling Zhu, Bing Wang, Zhi Zheng

**Affiliations:** 1 Department of Hepatopancreatobiliary Surgery, The Central Hospital of Wuhan, Tongji Medical College, Huazhong University of Science and Technology, Wuhan, Hubei, China; 2 Department of Thyroid and Breast Surgery, the Central Hospital of Wuhan, Tongji Medical College, Huazhong University of Science and Technology, Wuhan, Hubei, China; 3 Department of Hepatobiliary and Pancreatic Surgery, Tongji Hospital, Tongji Medical College, Huazhong University of Science and Technology, Wuhan, China

**Keywords:** choledocholithiasis, ERCP, laparoscopic, LCBDE, triple-endoscopic approach

## Abstract

**Background:**

Both endoscopic retrograde cholangiopancreatography (ERCP) and laparoscopic common bile duct exploration (LCBDE) have advantages and disadvantages. However, few surgeons integrate the strengths of both approaches to manage common bile duct stones.

**Materials and methods:**

We retrospectively analyzed the clinical data of 40 patients with gallbladder stones complicated by common bile duct stones, all of whom were treated by our treatment group between June 2024 and December 2024 in the Department of Hepatobiliary Surgery, Wuhan Central Hospital. Based on the surgical approach, the patients were divided into an experimental group (*n* = 30, combined use of laparoscopy, choledochoscopy, and duodenoscopy) and a control group (*n* = 10, ERCP followed by laparoscopic cholecystectomy). Data were collected in terms of demographic characteristics (age, gender, body mass index), intraoperative variables (operative time, estimated blood loss, and width of the common bile duct), and postoperative variables (time for postoperative gas passage, postoperative hospital stay, and complications).

**Results:**

In the experimental group, the mean age of the patients was 63 years. The mean operative time was 152 min. The mean estimated blood loss was 46 mL. Two patients (6.7%) suffered from postoperative pancreatitis. In one patient, the condition was caused by intraoperative stone extraction manipulation. The condition of the two patients improved after symptomatic treatment for pancreatitis was administered. One patient (3.3%) suffered from biliary fistula. Compared with the control group, hospital stay was significantly shorter in the experimental group; however, there was an increase in operative blood loss and operative time. No significant differences were found in the other indices.

**Conclusion:**

In the management of gallbladder stones with common bile duct stones, the “single-stage triple-endoscopic approach” represents a minimally invasive treatment with high clinical applicability. It is as safe and effective as traditional treatment regimens, and both ERCP and LCBDE have their own advantages.

## Introduction

Gallstone disease is highly common among adults (1). In general, few experience symptoms. Among those affected, 10%–25% may experience symptoms like biliary pain or acute cholecystitis, with 1%–2% developing serious complications (2, 3). Previous studies have reported that approximately 20% of patients with gallstones develop choledocholithiasis, leading to severe health consequences (4, 5). The typical clinical manifestations are obstructive jaundice and right upper quadrant pain, with more severe presentations involving pancreatitis or ascending cholangitis (6).

However, there is no clear consensus on the best management strategy for cholelithiasis. The treatment pathway often includes endoscopic retrograde cholangiopancreatography (ERCP) with laparoscopic cholecystectomy (LC) or laparoscopic common bile duct exploration (LCBDE) with LC (7). Many clinicians prefer an “endoscopy first” strategy, where ERCP is performed before LC, although this typically requires two separate general anesthesia procedures (8). Although ERCP is widely recognized for its diagnostic and therapeutic value, potential adverse events include pancreatitis, postsphincterotomy bleeding, infectious complications, and perforation (9). Although LCBDE preserves the function of the sphincter of Oddi and avoids the risks associated with endoscopic procedures, controversy remains regarding postoperative management—whether to place a T-tube or perform primary closure of the bile duct.

The purpose of this study is to share our experience on intraoperative combined application of laparoscopy, choledochoscopy, and duodenoscopy for the treatment of cholecystolithiasis complicated by choledocholithiasis.

## Materials and methods

We retrospectively analyzed the clinical data of 40 patients with gallbladder stones complicated by common bile duct stones, all of whom were treated by the same treatment group (all procedures were performed by the same chief surgeon, the corresponding author) between June 2024 and December 2024 in the department of Hepatobiliary Surgery, Wuhan Central Hospital. Based on the surgical approach, the patients were divided into an experimental group (*n* = 30, combined use of laparoscopy, choledochoscopy, and duodenoscopy) and a control group (*n* = 10, ERCP followed by LC). Data were collected in terms of demographic characteristics [age, gender, body mass index (BMI)], intraoperative variables [operative time, estimated blood loss (EBL), and width of the common bile duct], and postoperative variables (time for postoperative gas passage, postoperative hospital stay, and complications). Written consent was obtained from the patients associated in this study, and this study was permitted by the Ethics Committee of Wuhan Central Hospital.

### Statistical analysis

The software package SPSS 21.00 was utilized for the statistical analysis. The results were presented as numbers for categorical variables and as mean ± standard deviation for continuous percentage variables. For a comparison of the means of the groups, the Student’s *t*-test was utilized for the variables that demonstrated a normal distribution. A *p*-value < 0.05 was considered statistically significant.

### Inclusion criteria

1. Patients who were preoperatively diagnosed with gallbladder stones complicated by common bile duct stones based on imaging examinations such as CT and/or magnetic resonance cholangiopancreatography.
2. Patients who were preoperatively assessed to be able to tolerate anesthesia and surgery.
3. Patients who consented to surgical treatment after being informed of their condition, the relevant surgical precautions, and the associated risks.

### Exclusion criteria

1. Patients with concomitant intrahepatic bile duct stones.
2. Patients with concomitant primary biliary diseases such as gallbladder cancer, cholangiocarcinoma, or bile duct dilatation.
3. Patients in whom no gallbladder stones or common bile duct stones were found upon intraoperative exploration.
4. Patients with surgical contraindications such as severe cardiac or pulmonary diseases.

### Operative procedure

#### Experimental group

##### Patients’ position and trocar distribution

Patients were placed in supine position with reverse Trendelenburg and right-side elevation. In general, four trocars were used. The observing trocar (10 mm) was located at the inferior umbilicus. The primary trocar (12 mm) was located at the subxiphoid. Additional 5 mm trocars were located at the midline of the right collarbone and right axillary frontline. The surgeon and assistant stood on the right side of the patients.

### Operative procedure

**Figure 1:**
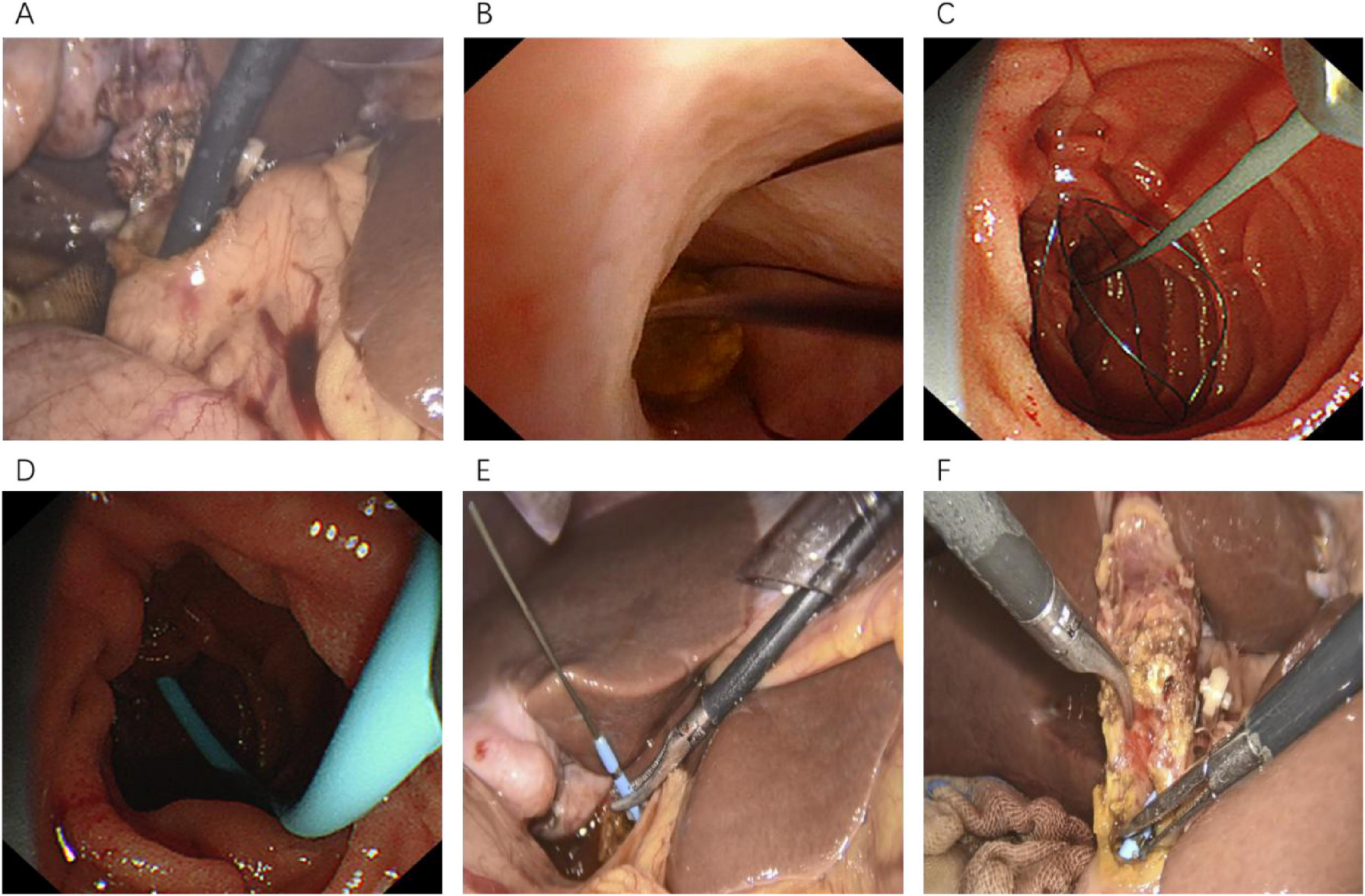
The operative procedure of intraoperative combined application of laparoscopy, choledochoscopy, and duodenoscopy for the treatment of cholecystolithiasis complicated by choledocholithiasis. **(A)** The choledochoscope enters the common bile duct. **(B)** The retrieval basket captured and removed the stones. **(C)** Retrieval of the guidewire with a basket. **(D)** The nasobiliary drainage tube is advanced over the guidewire into the common bile duct. **(E)** Placement of the nasobiliary drainage tube over the guidewire. **(F)** The nasobiliary drainage tube is placed into the common bile duct.

The patients underwent laparoscopic cholecystectomy under general anesthesia. First, the cystic artery and cystic duct were ligated, followed by cholecystectomy. Then, we performed a choledochotomy, inserted a choledochoscope to explore the bile ducts, and extracted stones using a basket catheter (Figures 1A, B). Following confirmation of patency in the distal common bile duct and the absence of residual stones via choledochoscopy, a basket retrieval catheter was introduced through the working channel of the choledochoscope, advanced through the duodenal papilla, and positioned within the lumen of the descending duodenum. The duodenoscope was advanced to the duodenal papilla. A Zebra guidewire was inserted into the duodenal lumen, and its tip was grasped with a basket retrieval catheter and then slowly withdrawn through the endoscope’s working channel (Figure 1C). We advanced the nasobiliary catheter over the guidewire, confirmed that its tip was in the common bile duct under fluoroscopy, injected sterile saline to check for kinking, and then withdrew the duodenoscope (Figures 1D, E). Finally, primary closure of the common bile duct was performed without T-tube placement (Figure 1F), and postoperative drainage was maintained via the nasobiliary catheter.

### Control group

ERCP was performed prior to LC.

## Result

The demographic characteristics of these patients are presented in Table 1. The experimental group included 17 female patients and 13 males. The mean age of these patients was 63 years (SD ± 15). The mean BMI was 23.3 kg/m^2^ (SD ± 3.0). The control group included four female patients and six males (*p* = 0.361). The mean age of these patients was 58 years (SD ± 18) (*p* = 0.390). The mean BMI was 23.5 kg/m^2^ (SD ± 3.5) (*p* = 0.860). There were no significant differences between the two groups in sex composition, mean age, or BMI.

**Table 1:** Demographic characteristics and the operative outcomes of patients.

Variables	Experimental group = 30	Control group = 10	*p*
Sex, male/female	13/17	6/4	0.361
Age, years	63 ± 15	58 ± 18	0.390
BMI, kg/m^2^	23.3 ± 3.0	23.5 ± 3.5	0.860
Operative time, min	152 ± 35	130 ± 30	0.042
EBL, mL	46 ± 15	32 ± 12	0.011
Width of the CBD, mm	12.8 ± 2.5	12.6 ± 2.8	0.830

The operative outcomes are presented in Table 1. No patient required conversion to open surgery. Our data showed that the operative time in the control group (the mean operative time was 130 min, 130 ± 30) was shorter than that in the experimental group (the mean operative time was 152 min, 152 ± 35) (*p* *<* *0.05*). In addition, the mean EBL was 46 mL (SD ± 15) in the experimental group and 32 mL (SD ± 12) in the control group (*p* < 0.05). There was no significant difference in the common bile duct diameter between the two groups (23.8 ± 2.5 vs. 12.6 ± 2.8 mm, *p* > 0.05).

The postoperative details of these patients are presented in Table 2. The median postoperative hospital stay was 11 days (SD ± 3) in the experimental group and 15 days (SD ± 4) in the control group (*p* *<* *0.05*). In addition, we observed no difference in the postoperative ventilation duration between the two groups (2.0 ± 0.8 vs. 2.3 ± 1.0 days, *p* > 0.05). Likewise, the rates of postoperative bile leakage and pancreatitis were low in both groups (*p* > 0.05). No patient suffered from perforation, intra-abdominal infection, and bleeding.

**Table 2:** Postoperative outcomes.

Variables	Experimental group = 30	Control group = 10	*p*
Post-operative hospital stay, days	11 ± 3	15 ± 4	0.002
Postoperative ventilation duration, days	2.0 ± 0.8	2.3 ± 1.0	0.340
Complications			
Bile leakage	1	0	0.617
Postoperative pancreatitis	2	0	1.000
Abdominal bleeding	0	0	
Delayed gastric emptying	0	0	
Perforation	0	0	
30-day mortality	0	0	

## Discussion

The concomitant presence of choledocholithiasis and cholecystolithiasis represents a clinically prevalent condition, accounting for approximately 6%–20% of all cholelithiasis cases. LC is the gold-standard surgical intervention for cholecystolithiasis. However, in cases complicated by concomitant choledocholithiasis, conventional management historically required open choledochotomy with stone extraction followed by T-tube drainage. LCBDE, along with LC, is being accepted as an appropriate treatment for common bile duct (CBD) stones. This approach demonstrates superior clinical efficacy as a single-stage intervention, characterized by significantly lower postoperative morbidity rates and reduced length of hospital stay compared with conventional multiprocedural strategies (7, 10). But this procedure requires expertise and instrumentation, and moreover, it significantly impairs patients’ postoperative quality of life (11).

Over the past decade, the evolution of minimally invasive surgical techniques has revolutionized the clinical management of cholecysto-choledocholithiasis (12). The advent of endoscopic management for common bile duct stones in 1974 marked a paradigm shift, rapidly establishing it as the criterion-standard intervention in biliary therapeutics (13, 14). Although contemporary ERCP demonstrates first-attempt success rates exceeding 90% at tertiary referral centers (15, 16), approximately 12%–15% of complex cases necessitate sequential endoscopic interventions to achieve complete ductal clearance (17). Despite being a relatively safe procedure, ERCP carries established procedural risks, including but not limited to postprocedural pancreatitis, gastrointestinal hemorrhage, and intestinal wall perforation (18). The therapeutic application of ERCP, combined with sphincterotomy, may compromise the anatomical integrity of the sphincter of Oddi, establishing a pathophysiological pathway for duodenobiliary reflux—a principal etiological factor in calculi reformation and persistent cholangitis (18, 19).

Subsequent decades have witnessed rigorous comparative analyses delineating procedure-specific outcomes, with particular focus on optimizing clinical decision-making pathways for choledocholithiasis management. Four clinical management strategies are currently available: preoperative ERCP followed by LC, laparoscopic cholecystectomy with concurrent intraoperative ERCP, laparoscopic cholecystectomy combined with intraoperative common bile duct exploration (CBDE), and postoperative ERCP performed after initial laparoscopic cholecystectomy.

LC + LCBDE are widely recognized as efficient, safe, and convenient, with high patient acceptance, as they enable the management of two separate pathological conditions in a single surgical intervention requiring only one anesthetic episode (20, 21). Laparoscopic exploration circumvents the potential complications of endoscopic sphincterotomy, such as pancreatitis, perforation, bleeding, cholangitis, and malignancies of the CBD. Choledochotomy may be followed by either direct closure of the CBD or closure with placement of a T-tube endoprosthesis. Nevertheless, the use of a T-tube is associated with potential complications such as accidental dislodgement, which may precipitate CBD obstruction, bile leakage, persistent biliary fistulas, skin excoriation, exogenous cholangitis, and fluid/electrolyte depletion. Furthermore, its requirement for extended and meticulous care constitutes a significant limitation to the patient’s quality of life. An additional single-procedure, minimally invasive strategy for cholecysto-choledocholithiasis is the performance of ERCP at the time of laparoscopic cholecystectomy (22, 23). Intraoperative ERCP can be conducted in several ways. Our team comprehensively integrated the strengths of LCBDE and ERCP and performed the one-stage approach—endoscopy approach with confirmation of thorough common bile duct stone clearance.

During the procedure, guidewire placement is achieved via antegrade choledochoscopic cannulation—advancing the wire through the CBD and traversing the papilla into the descending duodenal lumen. Compared with conventional ERCP retrograde cannulation, this method minimizes mechanical trauma to the duodenal papilla, facilitates endoscopic synergy—enabling seamless duodenoscopic/gastroscopic-guided retrieval of the guidewire, and simplifies drainage—allowing direct nasobiliary drainage catheter placement along the guidewire into the proximal CBD. In our study, postoperative pancreatitis occurred in two patients, which resolved with conservative management in both patients. Bile leakage is a common complication of LCBDE. However, a single case of postoperative mild biliary leakage was reported, which resolved completely with conservative management.

This study has several limitations. First, this study was a retrospective one, and the sample size was relatively small. A sufficiently powered, prospective randomized controlled trial is necessary to draw definitive conclusions. Second, the long-term outcomes of triple endoscopy combined surgery were not available. The long-term quality of life of patients, especially the incidence of delayed CBD structure, is important in this surgery.

## Conclusion

Theoretically, we suggest that the “one-stage approach” utilizing synchronous combined duodenoscopy, laparoscopy, and choledochoscopy offers advantages in terms of the incidence of postoperative acute pancreatitis, bile leakage rate, and length of hospital stay. However, since this is a retrospective clinical study with a small sample size, the findings obtained are limited. Therefore, large-sample, multicenter studies are needed in the future to further validate the reliability of this surgical approach. Nonetheless, we still consider the triple-scope combined procedure to be safe and effective for the treatment of gallbladder stones with common bile duct stones.

## Data Availability

The original contributions presented in the study are included in the article/supplementary material, and further inquiries can be directed to the corresponding author.
